# Exact Analytical Solution for 3D Time-Dependent Heat Conduction in a Multilayer Sphere with Heat Sources Using Eigenfunction Expansion Method

**DOI:** 10.1155/2014/708723

**Published:** 2014-11-17

**Authors:** Nemat Dalir

**Affiliations:** Department of Mechanical Engineering, Salmas Branch, Islamic Azad University, Salmas, Iran

## Abstract

An exact analytical solution is obtained for the problem of three-dimensional transient heat conduction in the multilayered sphere. The sphere has multiple layers in the radial direction and, in each layer, time-dependent and spatially nonuniform volumetric internal heat sources are considered. To obtain the temperature distribution, the eigenfunction expansion method is used. An arbitrary combination of homogenous boundary condition of the first or second kind can be applied in the angular and azimuthal directions. Nevertheless, solution is valid for nonhomogeneous boundary conditions of the third kind (convection) in the radial direction. A case study problem for the three-layer quarter-spherical region is solved and the results are discussed.

## 1. Introduction

Multilayer materials are composite media composed of several layers. Because of the additional benefit of combining various mechanical, physical, and thermal properties of different substances, a construction using multilayer elements is of interest. Multilayer materials are used in semicircular fiber insulated heaters, multilayer insulation materials, and nuclear fuel rods. Multilayer transient heat conduction finds applications in thermodynamics, fuel cells, and electrochemical reactors. The layered sphere is utilized to investigate the thermal properties of composite media by assuming embedded spherical particles in the composite matrix. For solving the problems of multilayer transient heat conduction, the same methods which are used in solving problems of single layer transient heat conduction are applied. These methods can be classified into two groups: analytical methods and numerical methods. Analytical methods are advantageous over numerical methods in two ways: (1) analytical solutions can be used as benchmark to examine and actually confirm numerical algorithms; (2) compared to a discrete numerical solution, the mathematical form of an analytical solution can provide better insight. It should also be mentioned that the analytical methods applied to multilayer transient conduction are analogous to those used in the single-layer transient heat conduction. These analytical methods include Green's function method, the Laplace transform, separation of variables, and eigenfunction expansion method.

Many researchers have solved the transient heat conduction problem in a composite medium. For instance, Salt [1] solved the transient heat conduction problem in a two-dimensional composite slab using an orthogonal eigenfunction expansion technique. Mikhailov and Özişik [2], using the orthogonal expansion approach, solved the problem of transient three-dimensional heat conduction in a composite Cartesian medium. Haji-Sheikh and Beck [3] used Green's function method to obtain temperature distribution in a three-dimensional two-layer orthotropic slab. de Monte [4, 5] applied the eigenfunction expansion method to obtain the transient temperature distribution for the heat conduction in a two-dimensional two-layer isotropic slab with homogenous boundary conditions. Lu et al. [6] and Lu and Viljanen [7] combined separation of variables and Laplace transforms to solve the transient conduction in the two-dimensional cylindrical and spherical media. Singh et al. [8, 9] and Jain et al. [10, 11] used the combination of separation of variables and eigenfunction expansion methods to solve the two-dimensional multilayer transient heat conduction in spherical coordinates. Recently, Dalir and Nourazar [12] used the eigenfunction expansion method to solve the problem of three-dimensional transient heat conduction in a multilayer cylinder.

Singh et al. [8, 9] and Jain et al. [10, 11] have studied 2D multilayer transient conduction problems in spherical and cylindrical coordinates. They have obtained analytical solutions for 2D multilayer transient heat conduction in spherical coordinates, in polar coordinates with multiple layers in the radial direction, and in a multilayer annulus. They have used the method of partial solutions to obtain the temperature distributions. In the method of partial solutions, the nonhomogeneous transient problem is split into two subproblems: a nonhomogeneous steady-state subproblem and a homogeneous transient subproblem. Then, the eigenfunction expansion method is used to solve the nonhomogeneous steady-state subproblem and the method of separation of variables is used to solve the homogeneous transient subproblem.

The literature survey for the exact analytical solution for 3D transient heat conduction in multilayered sphere demonstrates that such a solution has not, so far, been developed. Thus, in the present paper, using the eigenfunction expansion method, an analytical triple-series solution for transient heat conduction in the 3D spherical coordinates for radial multilayer domain with spatially nonuniform and time-dependent internal heat sources is obtained. Homogenous boundary conditions of the first or second kind can be applied on surfaces of *θ* = constant and *ϕ* = constant. However, nonhomogeneous boundary conditions of the third kind (convection) [11] are used in the *r*-direction.

Some assumptions are made for the 3D multilayer spherical transient conduction problem. First, the problem is a boundary-value problem of conduction in spherical (*r*-*θ*-*ϕ* coordinates) or part-spherical multilayer geometries. Second, volumetric internal heat sources of nonuniform and time-dependent (*r*, *θ*, *ϕ* and *t*-dependent) types are present. Third, on the inner and outer radial boundaries, nonhomogeneous boundary conditions of any kind can be used but, on the boundary surfaces in the *θ* and *ϕ*-directions, only the first or second kind of homogeneous boundary condition can be applied.

## 2. Mathematical Formulation

A *n*-layer composite spherical slab (*r*_0_ ≤ *r* ≤ *r*_*n*_, 0 ≤ *θ* ≤ *ψ*,  and  0 ≤ *ϕ* ≤ *ω*) is considered. All the layers have perfect thermal contact and are presumed to be isotropic in thermal properties. *α*_*i*_ and *k*_*i*_ are the temperature independent thermal diffusivity and thermal conductivity of the *i*th layer. At *t* = 0, the *i*th layer is at a specified temperature *f*_*i*_(*r*, *θ*, *ϕ*) and time dependent heat sources *g*_*i*_(*r*, *θ*, *ϕ*, *t*) are switched on in each radial layer. For *t* > 0, homogenous boundary conditions of first or second kind are applied to the angular surfaces of *θ* = 0 and *θ* = *ψ* and azimuthal surfaces of *ϕ* = 0 and *ϕ* = *ω*. For the inner (*i* = 1, *r* = *r*_0_) and the outer (*i* = *n*, *r* = *r*_*n*_) radial surfaces, all three kinds of boundary conditions are applicable.

The governing differential equation of the 3D transient conduction in a multilayered sphere with heat sources is as follows:

(1) ∂2Ti∂r2+2r∂Ti∂r+1r2∂2Ti∂θ2+cot⁡ θr2∂Ti∂θ+1r2sin2⁡θ∂2Ti∂ϕ2  +gir,θ,ϕ,tki=1αi∂Ti∂t,Ti=Tir,θ,ϕ,t, r0≤r≤rn,ri−1≤r≤ri, 1≤i≤n,0≤θ≤ψ, ψ<π,0≤ϕ≤ω,   ω<2π,t≥0.

The boundary conditions are as follows.

(i) Inner surface of 1st layer (*i* = 1):
  (2) $$\begin{array}{c} A_{\text{in}}\frac{\partial T_{1}}{\partial r}\left(r_{0},\theta ,\phi ,t\right)+B_{\text{in}}T_{1}\left(r_{0},\theta ,\phi ,t\right)=C_{\text{in}}. \end{array}$$
(ii) Outer surface of *n*th layer (*i* = *n*):
  (3) $$\begin{array}{c} A_{\text{out}}\frac{\partial T_{n}}{\partial r}\left(r_{n},\theta ,\phi ,t\right)+B_{\text{out}}T_{n}\left(r_{n},\theta ,\phi ,t\right)=C_{\text{out}}. \end{array}$$
(iii) *θ* = *ψ* surface (*i* = 1,2,…, *n*):
  (4) Tir,θ=ψ,ϕ,t=0 or ∂Ti∂θr,θ=ψ,ϕ,t=0,LLLLLLLLLLLLLLLLLLLLLLLLLLLi=1,…,n.
(iv) *ϕ* = 0 surface (*i* = 1,2,…, *n*):
  (5) Tir,θ,ϕ=0,t=0 or ∂Ti∂θ(r,θ,ϕ=0,t)=0,LLLLLLLLLLLLLLLLLLLLLLLLLLLi=1,…,n.
(v) *ϕ* = *ω* surface (*i* = 1,2,…, *n*):
  (6) Ti(r,θ,ϕ=ω,t)=0 or ∂Ti∂θ(r,θ,ϕ=ω,t)=0,LLLLLLLLLLLLLLLLLLLLLLLLLLLi=1,…,n.
(vi) Inner interface of the *i*th layer (*i* = 2,…, *n*):
  (7) $$\begin{array}{c} T_{i}\left(r_{i-1},\theta ,\phi ,t\right)=T_{i-1}\left(r_{i-1},\theta ,\phi ,t\right)\;i=2,\ldots ,n,\\ k_{i}\frac{\partial T_{i}}{\partial r}\left(r_{i-1},\theta ,\phi ,t\right)=k_{i-1}\frac{\partial T_{i-1}}{\partial r}\left(r_{i-1},\theta ,\phi ,t\right)\;i=2,\ldots ,n. \end{array}$$
(vii) Outer interface of the *i*th layer (*i* = 1,…, *n* − 1):
  (8) $$\begin{array}{c} T_{i}\left(r_{i},\theta ,\phi ,t\right)=T_{i+1}\left(r_{i},\theta ,\phi ,t\right)\;i=1,\ldots ,n-1, \end{array}$$
  (9) $$\begin{array}{c} k_{i}\frac{\partial T_{i}}{\partial r}\left(r_{i},\theta ,\phi ,t\right)=k_{i+1}\frac{\partial T_{i+1}}{\partial r}\left(r_{i},\theta ,\phi ,t\right)\;i=1,\ldots ,n-1. \end{array}$$

The initial condition is as follows:

(10) $$\begin{array}{c} T_{i}\left(r,\theta ,\phi ,t=0\right)=f_{i}\left(r,\theta ,\phi \right),\;1\leq i\leq n. \end{array}$$

It is worth mentioning that, at *r* = *r*_0_ and *r* = *r*_*n*_, boundary conditions of first, second, or third kind are applied by appropriate selection of the coefficients in (2) and (3). It should also be mentioned that zero inner radius (*r*_0_ = 0) for multilayered sphere is modeled by assigning zero values to *B*_in_ and *C*_in_ in (2) [8].

## 3. Solution Methodology

The eigenfunction expansion method is used to solve the problem. In the eigenfunction expansion method, first, by using the associated eigenvalue problem (∇^2^ *φ* = −*λ* ^2^ *φ*), the eigenfunctions are attained at every spatial direction of the problem. The associated eigenvalue problem is solved by the use of separation of variables. Afterward, the dependent variable and the available nonhomogeneity in the governing differential equation of the problem are separately written as series expansions of the eigenfunctions. In heat conduction problems, the dependent variable is temperature and the available nonhomogeneity is the volumetric heat source. The series expansions are then substituted into the differential equation. By performing some mathematical manipulations, an ordinary differential equation (ODE) is finally obtained for the independent variable. The solution of the problem is completed by solving this ODE, which is a first order ODE in the case of heat conduction problems.

As stated before, the method of partial solutions was used by Jain and Singh [11] for solving 2D transient heat conduction problems, the reason being that the heat source is independent of time. However, the method of partial solutions cannot be used for solving the present 3D transient heat conduction problem because the heat source depends on time. Thus, due to time dependence of the heat source, the partial solution of the steady-state subproblem cannot include the heat source term and the partial solutions method cannot be used. Therefore, to the best knowledge of the authors, the most efficient tool for solving the 3D heat conduction problem of the present paper is the eigenfunction expansion method.

For the transient problem of present paper, the associated eigenvalue problem is written as follows:

(11) ∇2φi=−λ2φi⟹∂2φi∂r2+2r∂φi∂r+1r2∂2φi∂θ2llllllllllllllilll+cot⁡ θr2∂φi∂θ+1r2sin2⁡θ∂2φi∂ϕ2llllllllllllll=−λ2φi.

Using the method of separation of variables, (11) is solved as follows:

(12) $$\begin{array}{c} \varphi_{i}\left(r,\theta ,\phi \right)=R_{i}\left(r\right)\Theta_{i}\left(\theta \right)\Phi_{i}\left(\phi \right), \end{array}$$

(13) Ri′′ΘiΦiRiΘiΦi+2rRi′ΘiΦiRiΘiΦi+1r2RiΘi′′ΦiRiΘiΦi+cot⁡ θr2RiΘi′ΦiRiΘiΦi  +1r2sin2⁡θRiΘiΦi′′RiΘiΦi=−λ2RiΘiΦiRiΘiΦi,

(14) $$\begin{array}{c} \frac{R_{i}^{\prime \prime }}{R_{i}}+\frac{2}{r}\frac{R_{i}^{\prime }}{R_{i}}+\frac{1}{r^{2}}\frac{\Theta_{i}^{\prime \prime }}{\Theta_{i}}+\frac{\text{cot}\;\theta }{r^{2}}\frac{\Theta_{i}^{\prime }}{\Theta_{i}}+\frac{1}{r^{2}\sin^{2}\theta }\frac{\Phi_{i}^{\prime \prime }}{\Phi_{i}}=-\lambda^{2}, \end{array}$$

(15) $$\begin{array}{c} \frac{R_{i}^{\prime \prime }}{R_{i}}+\frac{2}{r}\frac{R_{i}^{\prime }}{R_{i}}+\frac{1}{r^{2}}\frac{\Theta_{i}^{\prime \prime }}{\Theta_{i}}+\frac{\text{cot}\;\theta }{r^{2}}\frac{\Theta_{i}^{\prime }}{\Theta_{i}}=-\frac{1}{r^{2}\sin^{2}\theta }\frac{\Phi_{i}^{\prime \prime }}{\Phi_{i}}-\lambda^{2}, \end{array}$$

(16) sin2⁡θr2Ri′′Ri+2rRi′Ri+Θi′′Θi+cot⁡ θΘi′Θi+λ2r2  =−Φi′′Φi−=+ν2,

(17) $$\begin{array}{c} \Phi_{i}^{\prime \prime }+\nu_{im}^{2}\Phi_{i}=0⟶\Phi_{im}\left(\phi \right)=c_{1}\sin\nu_{im}\phi +c_{2}\cos\nu_{im}\phi , \end{array}$$

(18) ⟶r2Ri′′Ri+2rRi′Ri+λ2r2=−Θi′′Θi−cot⁡ θΘi′Θi+ν2sin2⁡θ=+β2,

(19) r2Ri′′+2rRi′+(λipn2r2−βn2)Ri=0  ⟶Ripn(r)=1rc3Jβn+0.5λipnrllllllllllllllllllllllllllllllllllllll+ c4Yβn+0.5λipnr,

By using the following change of variable:

(20) $$\begin{array}{c} \mu =\cos\theta ⟶1-\mu^{2}=\sin^{2}\theta \end{array}$$

the first and second derivatives of Θ with respect to *θ* are obtained as follows:

(21) Θ′=dΘidθ=dΘidμdμdθ=−sinθdΘidμ,Θi′′=d2Θidθ2=ddθdΘidθ=ddθ−sinθdΘidμ=−cos⁡θdΘidμ−sinθddθdΘidμ=sin2⁡θd2Θidμ2−cos⁡⁡θdΘidμ.

Substituting (21) in (19) results in the following:

(22) Θi′′+(cot⁡ θ)Θi′+β2−ν2sin2⁡θΘi=0  ⟶sin2⁡θd2Θidμ2−cos⁡θdΘidμ   +cos⁡θsinθ−sinθdΘidμ+β2−ν2sin2⁡θΘi=0  ⟶(1−μ2)d2Θidμ2−2μdΘidμ+βn2−νim21−μ2Θi=0.

If *β*_*n*_^2^ = *n*(*n* + 1), (22) is the associated Legendre equation. Its solution is written as follows:

(23) Θinμ=c5Pinνim(μ)+c6Qinνim(μ)⟶Θin(θ)=c5Pinνim(cos⁡θ)+c6Qinνimcos⁡θ→Qinνimcos⁡0 = Qinνim1 = ∞→c6 = 0Θinθ=Pinνimcos⁡θ.

The problem eigenvalues are *ζ*_*imnp*_^2^ = *λ*_*ipn*_^2^ + *ν*_*im*_^2^. It should be stated that the heat fluxes continuity at the interfaces of the radial layers gives the following:

(24) Φim=Φm,  νim=νm,λipn=λ1pnα1αi.

The eigenfunctions *R*_*ipn*_(*r*), Θ_*in*_(*θ*), and Φ_*im*_(*ϕ*) in *r*-, *θ*-, and *ϕ*-directions are derived by applying the boundary conditions in each direction in the following equation:

(25) $$\begin{array}{c} R_{ipn}\left(r\right)=\frac{1}{\sqrt{r}}\left[c_{3}J_{n\left(n\;+\;1\right)\;+\;0.5}\left(\lambda_{ipn}r\right)+c_{4}Y_{n\left(n\;+\;1\right)\;+\;0.5}\left(\lambda_{ipn}r\right)\right],\\ \Theta_{in}\left(\theta \right)=P_{in}^{\nu_{m}}\left(\cos\theta \right),\\ \Phi_{m}\left(\phi \right)=c_{1}\sin\nu_{m}\phi +c_{2}\cos\nu_{m}\phi . \end{array}$$

It is assumed that the solution of the problem is in the form of a triple-series expansion of the derived eigenfunctions as follows:

(26) $$\begin{array}{c} T_{i}\left(r,\theta ,\phi ,t\right)=\overset{\infty }{\underset{m\;=\;1}{\sum }}\overset{\infty }{\underset{n\;=\;1}{\sum }}\overset{\infty }{\underset{p\;=\;1}{\sum }}T_{imnp}\left(t\right)R_{ipn}\left(r\right)\Theta_{in}\left(\theta \right)\Phi_{m}\left(\phi \right). \end{array}$$

The heat source term is also written as a triple-series expansion of the eigenfunctions such that

(27) $$\begin{array}{c} g_{i}\left(r,\theta ,\phi ,t\right)=\overset{\infty }{\underset{m\;=\;1}{\sum }}\overset{\infty }{\underset{n\;=\;1}{\sum }}\overset{\infty }{\underset{p\;=\;1}{\sum }}g_{imnp}\left(t\right)R_{ipn}\left(r\right)\Theta_{in}\left(\theta \right)\Phi_{m}\left(\phi \right), \end{array}$$

where the coefficient *g*_*imnp*_(*t*) is obtained by the use of the orthogonality property as follows:

(28) gimnpt=∫0ω∫0ψ∫ri − 1rigi(r,θ,ϕ,t)r2llllllllllllllllllll×RipnrΘinθΦmϕdr dθ dϕ∫0ω ×∫0ω∫0ψ∫ri − 1rir2Ripn2rΘin2θlllllllllllllllllllllllll×Φm2ϕdr dθ dϕ∫0ω−1.

Substitution of (26) and (27) in (1) results in the following:

(29) Timnp(t)Ripn′′(r)Θin(θ)Φim(ϕ)+2rTimnp(t)Ripn′(r)Θin(θ)Φim(ϕ)+1r2Timnp(t)Ripn(r)Θin′′(θ)Φim(ϕ)  +cot⁡ θr2Timnp(t)Ripn(r)Θin′(θ)Φim(ϕ)+1r2sin2⁡θTimnptRipnrΘinθΦim′′ϕ  +1kigimnptRipnrΘinθΦimϕ=1αiTimnp′tRipnrΘinθΦimϕ,

(30) ⟶dTimnptdt+−αiRipn′′rRipnr+2rRipn′rRipnr+1r2Θin′′θΘinθ+cot⁡ θr2Θin′θΘinθ+1r2sin2⁡θΦim′′ϕΦimϕ︸= ΓimnpTimnpt=αikigimnpt.

Equation (30) is a first-order nonhomogeneous ODE and has the following solution:

(31) $$\begin{array}{c} T_{imnp}\left(t\right)=\frac{\alpha_{i}}{k_{i}}e^{-\Gamma_{imnp}t}\overset{\tau \;=\;t}{\underset{\tau \;=\;0}{\int }}g_{imnp}\left(\tau \right)e^{\Gamma_{imnp}\tau }d\tau +a_{i1}e^{-\Gamma_{imnp}t}. \end{array}$$

Application of the initial condition (10) on (26) gives the following:

(32) Tir,θ,ϕ,t=0  =fi(r,θ,ϕ)  =∑m = 1∞∑n = 1∞∑p = 1∞Timnp0RipnrΘinθΦimϕ.

The coefficient *a*_*i*1_ of (31) is found by the use of the orthogonality property for obtained eigenfunctions as follows:

(33) ai1=Timnp(0)=∫0ω∫0ψ∫ri−1rifir,θ,ϕr2RipnrΘinθΦimϕdr dθ dϕ∫0ω∫0ψ∫ri − 1rir2Ripn2rΘin2θΦim2ϕdr dθ dϕ.

The solution of differential equation (1) having (2) to (9) as boundary conditions and (10) as initial condition is (26) with (31) as the coefficients.

## 4. Case Study Problem

We consider a three-layer quarter-sphere (0 ≤ *r* ≤ *r*_3_,  0 ≤ *θ* ≤ *π*/2,  and  0 ≤ *ϕ* ≤ *π*) which is initially (*t* = 0) at uniform unit temperature. For time *t* > 0, thermal convection occurs, from the outer radial surface at *r* = *r*_3_ at (zero) ambient temperature. However, the *θ* = 0, *θ* = *π*/2, *ϕ* = 0, and *ϕ* = *π* surfaces are at uniform and constant zero temperatures. These boundary conditions lead to the following: *A*_in_ = 1, *A*_out_ = *k*_3_, *B*_in_ = 0, *B*_out_ = *h*, *C*_in_ = 0, and *C*_out_ = 0. Additionally, the uniformly distributed heat source *g*_*i*_, *i* = 1,…, 3, is turned on in each layer at *t* = 0. The governing differential equation for the 3D transient heat conduction with heat sources in this three-layer quarter-spherical region is as follows:

(34) ∂2Ti∂r2+2r∂Ti∂r+1r2∂2Ti∂θ2+cot⁡ θr2∂Ti∂θ+1r2sin2⁡θ∂2Ti∂ϕ2+giki  =1αi∂Ti∂t,Ti=Tir,θ,ϕ,t, 0≤r≤r3,ri−1≤r≤ri, 1≤i≤3,0≤θ≤π2,0≤ϕ≤π,t≥0.

The boundary conditions have the following forms:

(35) $$\begin{array}{c} \frac{\partial T_{1}}{\partial r}\left(0,\theta ,\phi ,t\right)=0, \end{array}$$

(36) $$\begin{array}{c} k_{3}\frac{\partial T_{3}}{\partial r}\left(r_{3},\theta ,\phi ,t\right)+hT_{3}\left(r_{3},\theta ,\phi ,t\right)=0, \end{array}$$

(37) $$\begin{array}{c} T_{i}\left(r,\frac{\pi }{2},\phi ,t\right)=0, \end{array}$$

(38) $$\begin{array}{c} T_{i}\left(r,\theta ,0,t\right)=0, \end{array}$$

(39) $$\begin{array}{c} T_{i}\left(r,\theta ,\pi ,t\right)=0. \end{array}$$

(i) Inner interface of the *i*th layer (*i* = 2,3):
  (40) $$\begin{array}{c} T_{i}\left(r_{i\;-\;1},\theta ,\phi ,t\right)=T_{i\;-\;1}\left(r_{i\;-\;1},\theta ,\phi ,t\right),\\ k_{i}\frac{\partial T_{i}}{\partial r}\left(r_{i\;-\;1},\theta ,\phi ,t\right)=k_{i-1}\frac{\partial T_{i\;-\;1}}{\partial r}\left(r_{i\;-\;1},\theta ,\phi ,t\right). \end{array}$$
(ii) Outer interface of the *i*th layer (*i* = 1,2):
  (41) $$\begin{array}{c} T_{i}\left(r_{i},\theta ,\phi ,t\right)=T_{i\;+\;1}\left(r_{i},\theta ,\phi ,t\right), \end{array}$$
  (42) $$\begin{array}{c} k_{i}\frac{\partial T_{i}}{\partial r}\left(r_{i},\theta ,\phi ,t\right)=k_{i\;+\;1}\frac{\partial T_{i\;+\;1}}{\partial r}\left(r_{i},\theta ,\phi ,t\right). \end{array}$$

The initial condition is as follows:

(43) $$\begin{array}{c} T_{i}\left(r,\theta ,\phi ,0\right)=1,\;1\leq i\leq 3. \end{array}$$

According to (25), by the use of the eigenfunction expansion method, the following solutions in *z*-, *r*-, and *θ*-directions are obtained from the associated eigenvalue problem:

(44) $$\begin{array}{c} \Phi_{m}\left(\phi \right)=c_{1}\sin\nu_{m}\phi +c_{2}\cos\nu_{m}\phi ,\\ \Theta_{in}\left(\theta \right)=P_{in}^{\nu_{m}}\left(\cos\theta \right),\\ R_{ipn}\left(r\right)=\frac{1}{\sqrt{r}}\left[c_{3}J_{n\left(n\;+\;1\right)\;+\;0.5}\left(\lambda_{ipn}r\right)+c_{4}Y_{n\left(n\;+\;1\right)\;+\;0.5}\left(\lambda_{ipn}r\right)\right]. \end{array}$$

The eigenvalues are *ζ*_*imnp*_^2^ = *λ*_*ipn*_^2^ + *ν*_*im*_^2^. The heat flux continuity conditions at the interfaces imply the following:

(45) $$\begin{array}{c} \lambda_{ipn}=\lambda_{1pn}\sqrt{\frac{\alpha_{1}}{\alpha_{i}}}. \end{array}$$

The eigenfunctions *R*_*ipn*_(*r*), Θ_*in*_(*θ*), and Φ_*m*_(*ϕ*) in the *r*, *θ*- and *ϕ*-directions are obtained by applying the relevant boundary conditions in each direction. Application of the boundary conditions in the *ϕ*-direction due to (44) gives the following:

(46) Φmϕ=c1sinνmϕ+c2cos⁡νmϕ⟶Φm(0)=0⟶c2=0 ⟶Φm(θ)=c1sinνmϕΦm(π)=0⟶c1sinνmπ=0 →c1 ≠ 0sinνmπ=0=sinmπ.

Then the *ϕ*-direction eigenvalues and eigenfunction are obtained as follows:

(47) $$\begin{array}{c} \nu_{m}=m,\;m=1,2,\ldots \\ \Phi_{m}\left(\phi \right)=\sin\left(m\phi \right). \end{array}$$

Using the *θ*-direction boundary condition, (37), on Θ in (44) results in the following relation:

(48) Θinθ=Pinmcos⁡θ→Θinπ/2 = 0Pinmcos⁡π2=0⟶Pinm(0)=0,

where *P*_*in*_^*m*^(0) = 0 is only satisfied when *n* are odd integers; that is, *n* = 1, 3, 5, …. Thus the *θ*-direction eigenvalues and the eigenfunction are as follows:

(49) $$\begin{array}{c} n=1,3,5,\ldots \\ \Theta_{in}\left(\theta \right)=P_{in}^{m}\left(\cos\theta \right). \end{array}$$

Applying the *r*-direction boundary conditions, that is, (35) and (36), gives the following:

(50) Ripnr  =1rc3Jnn + 1 + 0.5λipnr+c4Ynn + 1 + 0.5λipnr,lllllllllllllllllllllllllllllllllllllllllllllllllllllllllllllllllllli=1,2,3⟶R′(0)=0⟶R(0)=finite→Yn(n + 1) + 0.5(0) = ∞c4=0 ⟶Ripn(r)=c31rJn(n + 1) + 0.5(λipnr)kR′(r3)+hR(r3)=0 ⟶kc31r3Jn(n + 1) + 0.5′(λipnr3)   − kc312r3r3Jnn + 1 + 0.5(λipnr3)   + hc31r3Jn(n + 1) + 0.5(λipnr3)=0→c3 ≠ 01r3h−k2r3kJn(n + 1) + 0.5′(λipnr3)lllllllllllllllll+h−k2r3Jn(n + 1) + 0.5(λipnr3)=0⟶kJn(n + 1) + 0.5′(λipnr3)  +h−k2r3Jnn + 1 + 0.5(λipnr3)=0.

Thus the *r*-direction eigencondition and eigenfunction are derived as (*ν*_*m*_ = *m*):

(51) $$\begin{array}{c} kJ_{n(n\;+\;1)\;+\;0.5}^{\prime }\left(\lambda_{ipn}r_{3}\right)+\left(h-\frac{k}{2r_{3}}\right)J_{n(n\;+\;1)\;+\;0.5}\left(\lambda_{ipn}r_{3}\right)=0,\\ R_{ipn}\left(r\right)=\frac{1}{\sqrt{r}}J_{n\left(n\;+\;1\right)\;+\;0.5}\left(\lambda_{ipn}r\right). \end{array}$$

According to (28) to (33), the coefficients *g*_*imnp*_(*t*), *a*_*i*1_, and Γ_*imnp*_ are obtained as follows:

(52) gimnpt=∫0π∫0π∫ri − 1rigir21rJβn + 0.5λipnrPinmθllllllllllllllllllllll×sin⁡mϕdr dθ dϕ∫0π ×∫0π∫0π∫ri−1rirJβn + 0.52λipnrPinmθ2lllllllllllllllllllllllll×sin2⁡mϕdr dθ dϕ∫0π−1=∫0πgi∫ri−1rir3/2Jβn + 0.5λipnrdr∫0πPinmθdθllllll×∫0πsin⁡mϕdϕ ×∫ri−1rirJβn + 0.52λipnrdr∫0πPinmθ2dθlllllllll×∫0πsin2⁡mϕdϕ∫ri−1ri−1=−1mcos⁡⁡mθθ = 0θ = πgiμinmrJm + 1μinmrr = ri − 1r = rillllll× −1mcos⁡⁡mθθ = 0θ = π−1mcos⁡⁡mϕϕ = 0ϕ = π ×μinmr22Jm2μinmr+Jm+12μinmrr = ri − 1r = rillllllllll×π2×L2μinmr22−1=1−−1lgiμinmrJm + 1μinmrr = ri − 1r = ri×1m1−−1mllllll×Llπ1−−1l ×μinmr22Jm2μinmr+Jm+12μinmrr = ri − 1r = rillllllllll×πL4μinmr22−1⟶gimnp(t)=gimnp=ai1gi,Γimnp=−αi ×Ripn′′(r)Ripn(r)+1rRipn′(r)Ripn(r)+1r2Θin′′(θ)Θin(θ)+Φim′′(ϕ)Φim(ϕ)=−αiJm′′λipnrJmλipnr+1rJm′λipnrJmλipnr−m2r2−m2.

Using the form of a triple-series expansion of the obtained eigenfunctions, that is, (26), the solution is as follows:

(53) Tir,θ,ϕ,t=∑m = 1∞∑n = 1∞∑p = 1∞Timnpt1rJβn + 0.5llllllllllllllllllllllllllllllllllllll×λipnrPinmθsin⁡mϕ,

where the coefficient *T*_*imnp*_(*t*) is attained as follows:

(54) Timnpt=αikie−Γimnptgimnp1Γimnp(eΓimnpt−1)+ai1e−Γimnpt=αigimnpkiΓimnp+1gi−αikiΓimnpgimnpe−Γimnpt.

Therefore, the solution of (34) having (35) to (42) as boundary conditions and (43) as initial condition is (53) with (54) as the coefficients.

## 5. Conclusions

The exact analytical solution, that is, transient temperature distribution, is derived for the 3D transient heat conduction problem in a multilayered sphere using eigenfunction expansion method. Time-dependent and nonuniform volumetric heat generation is considered in each radial layer. Third kind nonhomogeneous boundary conditions are applied in the radial direction but the first or second kind homogenous boundary conditions are used in the angular and azimuthal directions. The heat conduction in a three-layer quarter-sphere is solved as a case study problem and the temperature distribution is found.

## Nomenclature

*A*_in_, *B*_in_, *C*_in_: Coefficients in (2)
*A*_out_, *B*_out_, *C*_out_: Coefficients in (3)
*f*_*i*_(*r*, *θ*, *ϕ*): Initial temperature distribution in the *i*th layer at *t* = 0
*g*_*i*_(*r*, *θ*, *ϕ*, *t*): Volumetric heat source distribution in the *i*th layer
*g*_*imnp*_: Coefficient in series expansion for heat source (Equation (27))
*h*: Outer surface heat transfer coefficient
*J*_*νm*_: Bessel function of the first kind of order *ν*_*m*_
*k*_*i*_: Thermal conductivity of the *i*th layer
*r*: Radial coordinate
*r*_*i*_: Outer radius for the *i*th layer
*R*_*ipn*_(*r*): Radial eigenfunctions for the *i*th layer
*t*: Time
*T*_*i*_(*r*, *θ*, *ϕ*, *t*): Temperature distribution for the *i*th layer
*T*_*imnp*_: Coefficient in general solution (Equation (26)) dependent on initial condition
*Y*_*νm*_: Bessel function of the second kind of order *ν*_*m*_.

### Greek Symbols

*α*_*i*_: Thermal diffusivity of the *i*th layer
*θ*: Azimuthal coordinate
Φ_*im*_(*ϕ*): Eigenfunctions in the *ϕ*-direction
Θ_*in*_(*θ*): Eigenfunctions in the angular direction
*λ*_*ipn*_: Radial eigenvalues
*ν*_*m*_: Eigenvalues in the *ϕ*-direction
*ω*: Angle subtended by the multilayers in the *ϕ*-direction
*ψ*: Angle subtended by the multilayers in the *θ*-direction
Γ_*imnp*_: Coefficient in (30).

### Subscripts and Superscripts

*i*: Layer or interface number
′: Differentiation.

## References

[1] Salt H. (1983). Transient heat conduction in a two-dimensional composite slab. I.Theoretical development of temperatures modes. *International Journal of Heat and Mass Transfer*.

[2] Mikhailov M. D., Özişik M. N. (1986). Transient conduction in a three-dimensional composite slab. *International Journal of Heat and Mass Transfer*.

[3] Haji-Sheikh A., Beck J. V. (2002). Temperature solution in multi-dimensional multi-layer bodies. *International Journal of Heat and Mass Transfer*.

[4] de Monte F. (2004). Transverse eigenproblem of steady-state heat conduction for multi- dimensional two-layered slabs with automatic computation of eigenvalues. *International Journal of Heat and Mass Transfer*.

[5] de Monte F. (2006). Multi-layer transient heat conduction using transition time scales. *International Journal of Thermal Sciences*.

[6] Lu X., Tervola P., Viljanen M. (2006). Transient analytical solution to heat conduction in composite circular cylinder. *International Journal of Heat and Mass Transfer*.

[7] Lu X., Viljanen M. (2006). An analytical method to solve heat conduction in layered spheres with time-dependent boundary conditions. *Physics Letters Section A: General, Atomic and Solid State Physics*.

[8] Singh S., Jain P. K., Rizwan-uddin (2008). Analytical solution to transient heat conduction in polar coordinates with multiple layers in radial direction. *International Journal of Thermal Sciences*.

[9] Singh S., Jain P. K. (2011). Finite integral transform method to solve asymmetric heat conduction in a multilayer annulus with time-dependent boundary conditions. *Nuclear Engineering and Design*.

[10] Jain P. K., Singh S., Rizwan-uddin (2009). Analytical solution to transient asymmetric heat conduction in a multilayer annulus. *Journal of Heat Transfer*.

[11] Jain P. K., Singh S. (2010). An exact analytical solution for two-dimensional, unsteady, multilayer heat conduction in spherical coordinates. *International Journal of Heat and Mass Transfer*.

[12] Dalir N., Nourazar S. S. (2014). Analytical solution of the problem on the three-dimensional transient heat conduction in a multilayer cylinder. *Journal of Engineering Physics and Thermophysics*.

